# Survival and prognostic factors in vaginal cancer: an analysis of 2016–2020 nationwide data

**DOI:** 10.1007/s10147-026-03017-y

**Published:** 2026-04-09

**Authors:** Tatsuhiko Shigeto, Yoshihito Yokoyama, Wataru Yamagami, Fumiaki Takahashi, Hideki Tokunaga, Eiko Yamamoto, Kiyoshi Yoshino, Kei Kawana, Satoru Nagase

**Affiliations:** 1 Department of Obstetrics and Gynecology, Hirosaki University Graduate School of Medicine, 5 Zaifu-Cho, Hirosaki, Aomori 036-8562 Japan; 2 Department of Obstetrics and Gynecology, Keio University School of Medicine, Tokyo, Japan; 3 Department of Information Science, Iwate Medical University, Iwate, Japan; 4 Devision of Obstetrics and Gynecology, Tohoku Medical and Pharmaceutical University, Miyagi, Japan; 5 Department of Healthcare Administration, Nagoya University Graduate School of Medicine, Nagoya, Japan; 6 Department of Obstetrics and Gynecology, University of Occupational and Environmental Health, Fukuoka, Japan; 7 Department of Obstetrics and Gynecology, Nihon University School of Medicine, Tokyo, Japan; 8 Department of Obstetrics and Gynecology, Yamagata University, Yamagata, Japan

**Keywords:** Vaginal cancer, Surgery, Radiation therapy, Prognosis, Registries

## Abstract

**Background:**

Vaginal cancer is a rare gynecological malignant disease. In Japan, the proportion of vaginal cancer cases is estimated to increase as the population ages. The purpose of this study is to evaluate the age-specific incidence, treatment options, and prognosis of vaginal cancer in Japan.

**Methods:**

This retrospective observational study used the Japan Society of Obstetrics and Gynecology tumor registry database. Patient characteristics included age, year diagnosed with vaginal cancer (2016–2020), and cancer stages, histological types, and treatment methods. The 2016 and 2017 dataset included follow-up time, survival status, and cause of death.

**Results:**

A total of 746 patients were diagnosed with vaginal cancer from 2016 to 2020. The median age of the patients was 72 years. Radiotherapy was most chosen as the primary treatment option for all ages and stages. Surgery was selected mostly in stage I and II (*n* = 118, 22.2% of these stage patients). Five-year overall survival for stages I, II, III, and IV was 87.6%, 55.6%, 59.9%, and 15.3%, respectively. Multivariate analysis showed that 3 year OS was significantly better for patients who underwent surgery than those who received chemotherapy (HR 3.11, *P* = 0.00389) and those who received radiation therapy (HR 2.89, *P* = 0.0241), but not compared with concurrent chemoradiotherapy (HR 1.21, *P* = 0.7091). Histological type did not significantly affect prognosis. For stages I and II, surgery resulted in a better prognosis than radiation (*P* = 0.03).

**Conclusions:**

Radiation therapy is the primary treatment for vaginal cancer; however, surgery may be an effective option in early-stage vaginal cancer when cases are appropriately selected.

**Supplementary Information:**

The online version contains supplementary material available at 10.1007/s10147-026-03017-y.

## Introduction

Vaginal cancer is a rare malignant disease, approximately accounting for 1%–3% of all gynecologic malignancies [1]. The dataset indicated that in the USA, an estimated 8070 new cases of vaginal and other genital cancers would be diagnosed in 2025, and 1950 people were estimated to die of the disease [2]. Because of the rarity of this disease, there is still limited information on its epidemiology, treatment, survival, and prognostic factors. Vaginal cancer is most common in women aged 70 and older. Age-standardized incidence rate of vaginal preinvasive neoplasia ranges between 0.5 to 1.3 per 100,000 women and those aged 60–69 are at greater risk [3]. The majority of invasive vaginal tumors are squamous cell carcinoma (SCC, 79–85% of cases), followed by adenocarcinoma (5–14%) [5], melanoma (1–5%) [6] and sarcoma. Vaginal cancer most commonly arises in the upper portion of the posterior wall of the vagina. The retrospective analysis of 91 patients showed that the tumor involved the upper third of the vagina in 46.2% of the cases [7]. Risk factors for vaginal cancer based on etiologic insights from case studies include vaginal damage from ring pessaries, chronic vaginitis, high sexual activity, tobacco usage, and HPV infection [8, 9]. Some of these risk factors overlap with those for cervical cancer.

Although there is no standardized treatment for this rare cancer, surgery and radiation are the main treatments. According to the NCCN guidelines, primary treatment for early-stage vaginal cancer is radiation therapy (RT) and surgical resection. For the majority of vaginal cancers, RT is preferred rather than surgery as primary treatment due to improved organ preservation. For invasive stage II–IVA disease, the preferred treatment is platinum-based chemoradiation therapy (CCRT) with brachytherapy. For stage IVB patients, systemic therapy is one of the options. Local treatment of the primary disease by chemoradiation with/without brachytherapy can be considered [10].

In Japan, estimated incidence figures are available in the National Cancer Center database. However, nationwide incidence data for vaginal cancer were lacking until 2016, because accurate case numbers, treatments, and prognoses were not collected. The Japanese Society of Obstetrics and Gynecology (JSOG) included vaginal cancer alongside other rare gynecologic malignancies such as vulvar cancer, uterine sarcoma, and chorionic carcinoma in its tumor registry. Nationwide data collection began in 2016, following cervical, endometrial, and ovarian cancers. However, there are still few reports on treatment choices and prognostic trends for vaginal cancer in Japan. Japan is one of the world's most rapidly aging nations [11], making the analysis of vaginal cancer, which is frequently observed in the elderly, particularly important. This study is the first to evaluate the trends for treatment options and the prognosis of vaginal cancer in Japan.

## Patients and methods

### Data extraction

This retrospective observational study used the Japan Society of Obstetrics and Gynecology (JSOG) database, a publicly available and deidentified database, with prior approval. The JSOG tumor registry database is a national, hospital-based gynecologic cancer registry launched in 2001, supported and managed by the gynecologic tumor committee of the JSOG. All participating centers adhered to the same definition and submitted the data annually to the JSOG committee using the same online format. Within the extracted dataset, the study population included women with vaginal cancer who had undergone initial treatment from 2016 to 2020. The Ethics Committee of Keio University and the JSOG, which have authority to approve studies conducted using JSOG tumor registry data, approved this study.

Clinicopathological details included age, year diagnosed with vaginal cancer (2016–2020), cancer stage, histological type, and treatment method. Cancer stages are classified based on the 1971 International Federation of Gynecology and Obstetrics (FIGO) staging system, and the Union for International Cancer Control (UICC) TNM classification 7th edition. The TNM classification of the cases in which surgery was not performed as initial treatment (e.g., radiation or chemotherapy) was estimated by imaging diagnosis. Histological types included squamous cell carcinoma, adenocarcinoma, adenosquamous carcinoma, and others. Treatment types included primary treatment type (surgery, chemotherapy, radiation therapy, CCRT, and others), and adjuvant treatment. The 2016 and 2017 dataset included prognostic information whether the patients were alive and the last date of confirmed survival. The 2017 dataset recorded only the status at 3 years after the primary treatment.

### Statistical analysis

Survival curves were generated using the Kaplan–Meier method, and the difference between the curves was assessed using the log-rank test. The univariate and multivariate analyses were used to evaluate case survival factors. The Cox proportional hazard regression model was used for the analysis, and the effect size was expressed as hazard ratio with 95% CI. All statistical analyses were based on a two-sided hypothesis, with *p* < 0.05 considered statistically significant. All statistical analyses were performed with EZR (Saitama Medical Center, Jichi Medical University, Saitama, Japan), which is a graphical user interface for R (The R Foundation for Statistical Computing, Vienna, Austria) [12].

## Result

### Patient demographics

Table 1 shows the demographic and clinicopathological characteristics. A total of 746 patients were diagnosed and registered with vaginal cancer. The median age of the patients was 72 years. The ages ranged 1 to 95 years. Age distribution of patients with vaginal cancer was as follows: 77 (10.3%), 245 (32.8%), 210 (28.2%), and 214 (28.7%) patients belonged to the under 50, 50–69, 70–79, and 80 or higher age groups, respectively. Cancer stages were I: 42.5%, II: 28.8%, III: 12.2%, and IV: 16.5%. Histological types were squamous cell carcinoma: 79.6%, adenocarcinoma: 8.2%, adenosquamous carcinoma: 1.5%, and other: 10.1%. As primary treatment, 22.9% of patients underwent surgery, 6.0% received chemotherapy, 43.4% received radiation therapy alone, and 26.8% received CCRT. In addition, of the 171 patients who underwent surgery as the primary treatment, 26 (15.2%) received chemotherapy, 21 (12.3%) received RT, 20 (11.7%) received CCRT, and 3 (1.7%) received other treatments; 101 (59.1%) patients were treated with surgery alone.

**Table 1:** Demographic and clinicopathological characteristics

Variables	Number	%
Total	746	100
Age (years, range:1–95, medium:72)		
< 50	77	10.3
50–69	245	32.8
70–79	210	28.2
≥ 80	214	28.7
FIGO stage		
Ⅰ	317	42.5
Ⅱ	215	28.8
Ⅲ	91	12.2
Ⅳ	123	16.5
TMN		
T		
T0	1	0.1
Tis	7	0.9
T1	315	42.2
T2	236	31.6
T3	89	11.9
T4	90	12.1
TX	8	1.1
N		
N0	425	57.0
N1	206	27.6
NX	115	15.4
M		
M0	662	88.7
M1	73	9.8
MX	11	1.5
Histology		
Squamous cell carcinoma	594	79.6
Adenocarcinoma	61	8.2
Adenosquamous carcinoma	11	1.5
Other	75	10.1
Unknown	5	0.7
Primary treatment		
Surgery	171	22.9
Chemotherapy	45	6.0
Radiation	324	43.4
CCRT	200	26.8
Other	6	0.8
ypTMN		
ypT		
T0	2	0.3
Tis	0	0.0
T1	6	0.8
T2	3	0.4
T3	1	0.1
T4	2	0.3
TX	0	0.0
ypN		
N0	8	1.1
N1	3	0.4
NX	3	0.4
ypM		
M0	12	1.6
M1	1	0.1
MX	1	0.1

### Treatment options by stages and ages

Radiation therapy was mostly chosen as the primary treatment option across the entire patients (Table 2). Among all patients, 43.4%, 26.8%, 22.9%, and 6.0% received radiation therapy alone, CCRT, surgery, and chemotherapy as a primary treatment, respectively. Most surgeries were performed in stages I and II. Evaluating treatment options by ages, surgery was most chosen in the < 50 age group, while radiation therapy was most chosen in the ≥ 80 age group (Table 3). The incidence of advanced cancer tended to increase with age. Stage III and IV patients accounted for only 20.7% (16/77) in the < 50 years group, but they accounted for 31.2% (67/215) in the ≥ 80 years group (Table 4).

**Table 2:** Number of primary treatments by stage

	Stage	I	II	III	IVA	IVB	Total
Primary treatment							
Surgery		118 (15.9%)	36 (4.8%)	3 (0.4%)	6 (0.8%)	8 (1.1%)	171 (22.9%)
Chemotherapy		7 (1.0%)	6 (0.8%)	7 (1.0%)	4 (0.5%)	21 (2.9%)	45 (6.0%)
Radiation		143 (19.2%)	94 (12.6%)	37 (5.0%)	22 (3.9%)	28 (3.8%)	324 (43.4%)
CCRT		48 (6.4%)	78 (10.5%)	43 (5.8%)	17 (2.3%)	14 (18.8%)	200 (26.8%)
Other		1 (0.1%)	1 (0.1%)	1 (0.1%)	1 (0.1%)	2 (0.3%)	6 (0.8%)
Total		317 (42.4%)	215 (28.8%)	91(12.2%)	50 (6.7%)	73 (9.8%)	746 (100%)

*CCRT* concurrent chemoradiation therapy

**Table 3:** Number of primary treatments by age

	Age	< 50	50–69	70–79	≥ 80	Total
Primary treatment						
Surgery		30 (4.0%)	96 (12.9%)	30 (4.0%)	15 (2.0%)	171 (22.9%)
Chemotherapy		5 (0.7%)	16 (2.1%)	15 (2.0%)	9 (1.2%)	45 (6.0%)
Radiation		12 (1.6%)	47 (6.3%)	91 (12.0%)	174 (23.3%)	324 (43.4%)
CCRT		30 (4.0%)	84 (11.3%)	73 (9.8%)	13 (1.7%)	200 (26.8%)
Other		0	1 (0.1%)	1 (0.1%)	4 (0.5%)	6 (0.8%)
Total		77 (10.3%)	244 (32.7%)	210 (28.1%)	215 (28.8%)	746 (100%)

*CCRT* concurrent chemoradiation therapy

**Table 4:** FIGO stage and age distribution

	Age	< 50	50–69	70–79	≥ 80	Total
FIGO stage						
I		42 (5.6%)	117 (22.8%)	81 (10.9%)	77 (10.3%)	317 (42.4%)
II		19 (2.5%)	64 (9.0%)	61 (8.2%)	71 (9.5%)	215 (28.8%)
III		9 (1.2%)	23 (3.1%)	29 (3.9%)	30 (4.0%)	91(12.2%)
IV		7 (0.9%)	40 (5.7%)	39 (5.2%)	37 (4.0%)	123 (16.5%)
IVA		3 (0.4%)	16 (0.8%)	16 (2.1%)	15 (2.0%)	50 (6.7%)
IVB		4 (0.5%)	24 (0.3%)	23 (3.1%)	22 (3.0%)	73 (9.8%)
Total		77 (10.3%)	244 (32.7%)	210 (28.2%)	215 (28.8%)	746 (100%)

### Prognosis of registered patients in 2016 and 2017

**Fig. 1:**
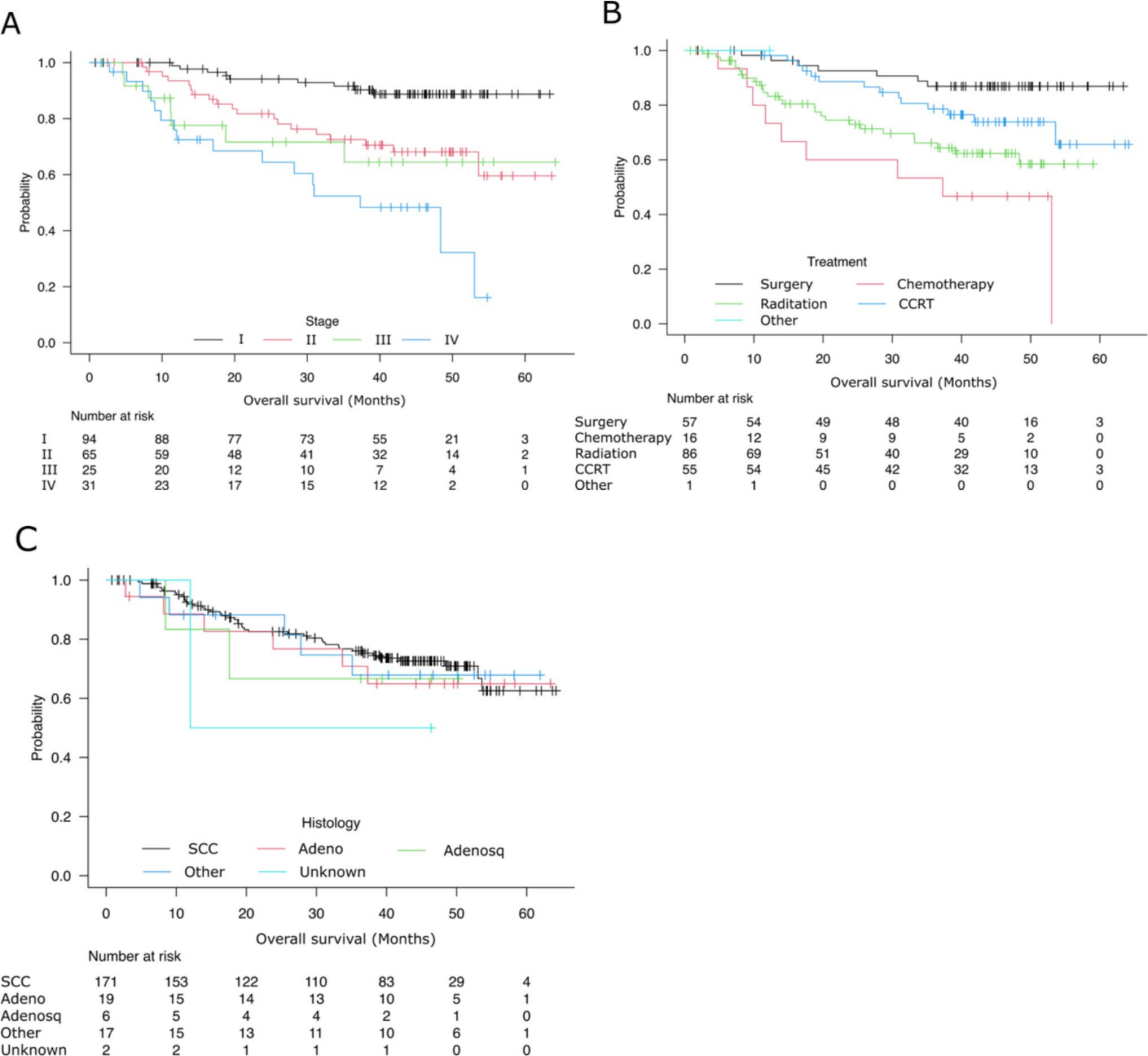
Survival curves for 215 patients with vaginal cancer. Overall survival was analyzed by stage (**A**), treatment methods (**B**), and histologies (**C**). *SCC* squamous cell carcinoma, *Adenosq* adenosquamous carcinoma, *Adeno* adenocarcinoma, *CCRT* concurrent chemoradiation therapy

In 2016 and 2017, 260 patients with vaginal cancer were registered, and 215 patients had prognostic information. Forty-five (17.3%) patients were lost to follow-up. Based on the Kaplan–Meier curved by stage, the 5 year overall survival rate for stages I, II, III, and IV were 87.6%, 55.6%, 59.9%, and 15.3%, respectively (Fig. 1A). The median follow-up period for these patients was 1181 days, with a range from 24 to 1925 days. There was only 3-year prognostic information in the 2017 database; univariate and multivariate analysis were performed for the 3 year overall survival rate (Table 5). The survival curve for each treatment is shown in Fig. 1-B. Univariate analysis showed that FIGO stage and chemotherapy were related to prognosis. 3 year OS gradually decreased as age increased (< 50 years: 82.6%, 50–69 years: 79.3%, 70–79 years: 75.3%, ≥ 80 years: 60.7%); however, no statistical significance was confirmed. Three-year OS was significantly better for patients who underwent surgery (86.9%) than those who received chemotherapy (53.5%; HR 3.13, *P* = 0.004). In multivariate analysis, age ≥ 80 years, FIGO stage, chemotherapy, and radiation were independent prognostic factors. Patients aged < 50 years had lower mortality risks than patients aged ≥ 80 years (HR: 2.92, 95%CI 2.92–7.96, *P* = 0.035). Three-year OS was significantly better for patients who underwent surgery than those who received chemotherapy (HR 3.11, *P* = 0.0389) and those who received radiation therapy (HR 2.89, *P* = 0.0241). However, no significant difference was observed between surgery and CCRT (HR 1.21, *P* = 0.7091). As expected, FIGO stage was strongly related to prognosis, as patients with stage I disease had lower mortality risks than those with stage II (HR: 3.50, *P* = 0.0022), stage III (HR: 5.29, *P* = 0.0007), and stage IV (HR: 6.01. *P* = 0.0001). Histological type did not significantly affect prognosis. The survival curve is shown in Fig. 1C.

**Table 5:** Univariate and multivariate survival analysis

Variables	*n*(%)	3 year OS	Univariate analysis		Multivariate analysis	
			HR(95% CI)	*P*	HR(95% CI)	*P*
Age						
< 50	24 (11.2)	82.6%	Reference		Reference	
50–69	85 (39.5)	79.3%	1.02 (0.36–2.858)	0.9708	1.06 (0.40–2.87)	0.9019
70–79	53 (24.7)	75.3%	1.50 (0.51–4.41)	0.4657	1.81 (0.66–4.93)	0.2500
≥ 80	53 (24.7)	60.7%	1.78 (0.56–5.71)	0.3305	2.92 (2.92–7.90)	0.0348
FIGO stage						
Ⅰ	94 (54.7)	90.4%	Reference		Reference	
Ⅱ	65 (30.2)	67.7%	3.03 (1.35–6.78)	0.0071	3.50 (1.57–7.82)	0.0022
Ⅲ	25 (11.6)	59.9%	4.78 (1.79–12.80)	0.0018	5.29 (1.93–14.5)	0.0007
Ⅳ	31 (14.4)	49.7%	5.46 (2.31–12.92)	0.0001	6.01 (2.51–14.38)	0.0001
Histology						
SCC	171 (79.5)	76.0%	Reference		Reference	
Adeno	19 (8.8)	70.8%	1.49 (0.59–3.79)	0.3940	1.29 (0.52–3.22)	0.5838
Adenosq	6 (2.8)	66.7%	1.94 (0.43–8.70)	0.3846	1.75 (0.39–7.81)	0.4649
Other	17 (7.9)	67.9%	1.26 (0.46–3.42)	0.6464	1.24 (0.46–3.33)	0.6722
Unknown	2 (0.9)	50.0%	1.36 (0.18–11.1)	0.7723	1.30 (0.16–10.41)	0.8043
Primary treatment						
Surgery	57 (26.5)	86.9%	Reference		Reference	
Chemotherapy	16 (7.4)	53.3%	3.13 (1.04–9.43)	0.0042	3.11 (1.06–9.12)	0.0389
Radiation	86 (40.0)	66.1%	2.30 (0.89–5.95)	0.0849	2.89 (1.15–7.25)	0.0241
CCRT	55 (25.6)	78.6%	1.27 (0.46–3.25)	0.6455	1.21 (0.44–3.36)	0.7091
Other	1 (0.5)	100.0%	0.00(-)	0.9962	0.00 (-)	0.9962

Cox Regression Analysis

*SCC* squamous cell carcinoma, *Adeno* adenocarcinoma, *Adenoaq* adenosquamous carcinoma, *CCRT* concurrent chemoradiation therapy, *HR* hazard ratio

Surgery was selected as the primary treatment in 57 (26.5%) cases, of which 43 were selected in stage I, 9 in stage II, 2 in stage III, and 3 in stage IV.

**Fig. 2:**
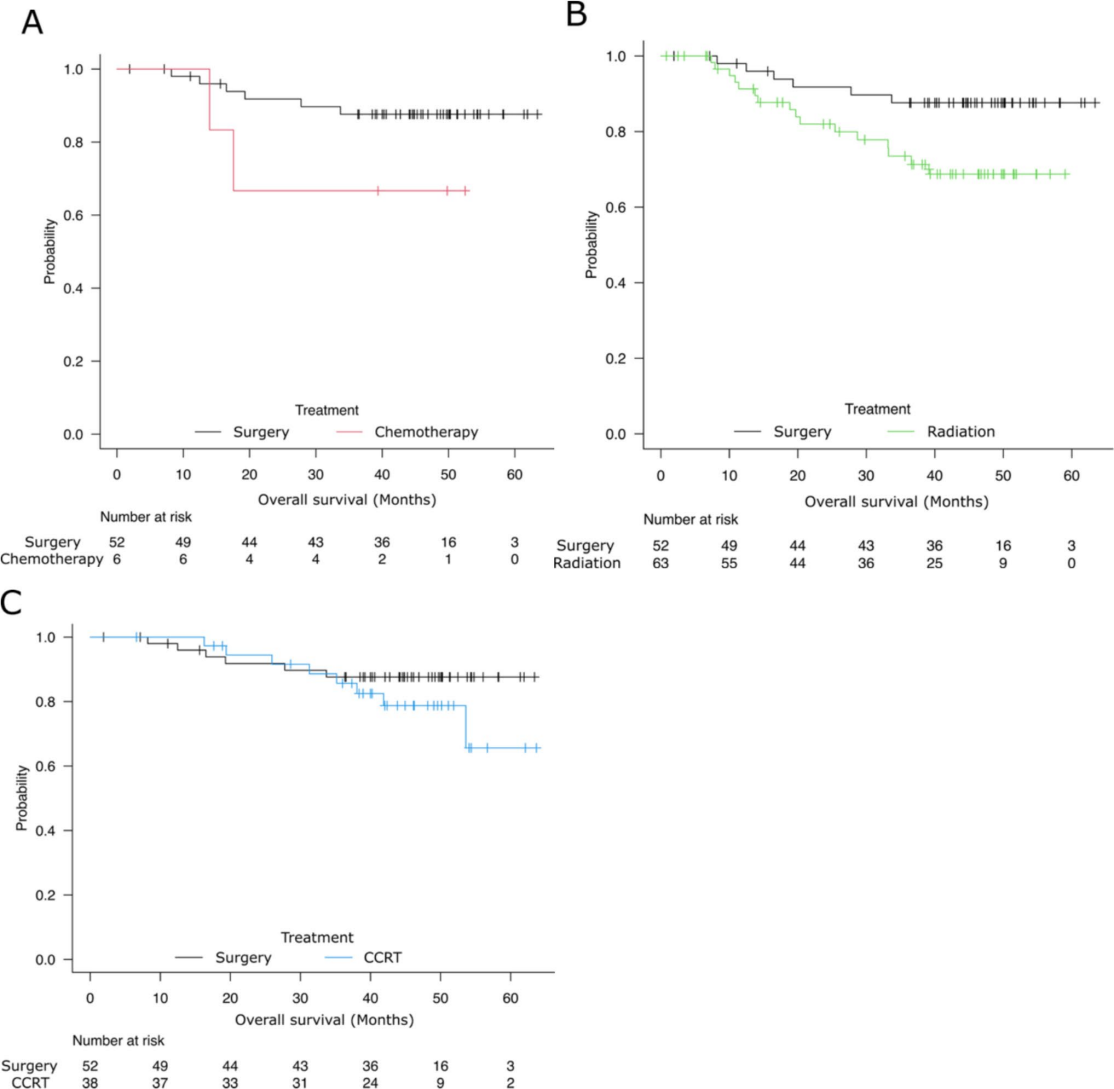
Comparison of prognosis with surgery in stage I and II patients. **A** Surgery vs*.* chemotherapy (*P* = 0.10), **B** surgery vs*.* radiation (*P* = 0.03), **C** surgery vs. CCRT (*P* = 0.25). *CCRT* concurrent chemoradiation therapy

For stages I and II, the surgery group had a better prognosis than the radiation group (Fig. 2B *P* = 0.030). No significant difference was observed between the surgery group and the chemotherapy group, or between the surgery group and the CCRT group (Fig. 2A, C). However, limiting to stage I, there was no significant difference in prognosis among these four treatment groups (Supplementary Fig.  1 A). Among the surgery group, 9 of 43 patients received radiation therapy including CCRT, and 2 patients received chemotherapy after surgery. Compared to the surgery-only group, the group receiving postoperative radiation therapy (surgery plus RT group) had a tendency toward poorer prognosis; however, no significant difference was observed (Supplementary Fig. 1-B, *P* = 0.065). Additionally, there was no significant difference between the surgery plus RT group and the radiation group (*P* = 0.764), or between the surgery plus RT group and CCRT (*P* = 0.765).

For stage II patients, CCRT had a tendency toward better prognosis, but no significant difference was observed compared to other groups (Supplementary Fig. 2). Among the surgery group, six of nine patients received adjuvant therapy. Four patients received radiation therapy or CCRT, and two patients received chemotherapy after surgery.

**Fig. 3:**
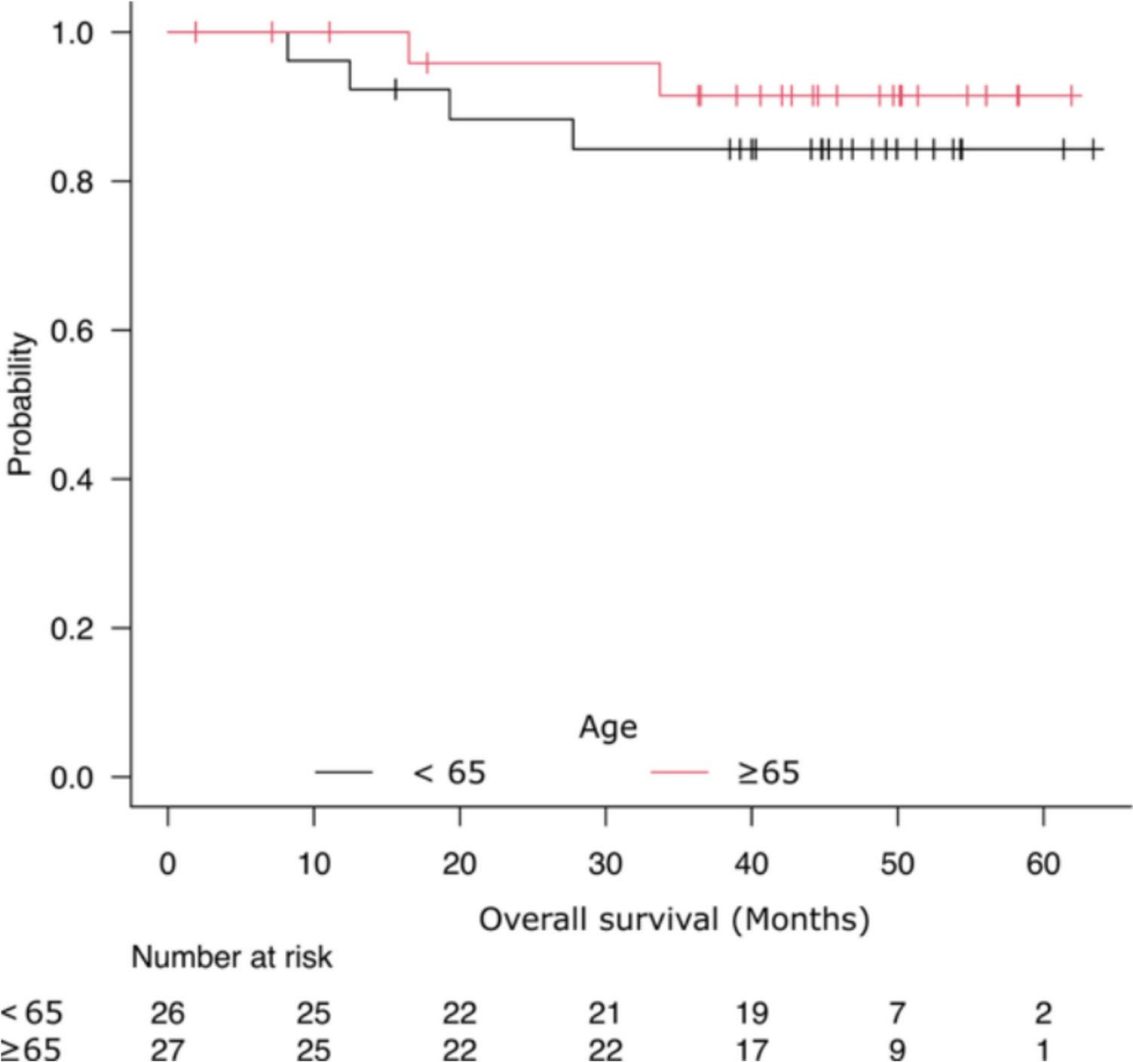
Comparison of prognosis by age in surgical cases. There was no difference in prognosis according to age (*P* = 0.420)

Furthermore, when the surgical cases were divided into less than 65 years of age and those 65 years of age or older age group, there was no significant difference in prognosis according to age (Fig. 3).

## Discussion

Vaginal cancer is a rare gynecological malignant disease. Only a few retrospective studies of this disease have been published, and clinical information is very limited.

Although vaginal cancer shares some of the same risk factors as cervical cancer, it is more likely to occur among older women, compared with cervical cancer. The median age for individuals diagnosed with invasive cervical cancer was 47 years, while for those with invasive vaginal cancer it was 68 years [13]. The incidence of primary vaginal cancer increases with age, with approximately 50% of patients diagnosed at age ≥ 70, and 20% at age ≥ 80 [14]. This aligns with our findings where 56.9% patients were registered at age ≥ 70 and 28.7% at age ≥ 80. Squamous cell carcinoma is the most common histological type of vaginal cancer. It is reported to account for 79–85% of vaginal cancer, followed by adenocarcinoma (5–14%) and melanoma (1–5%) [4]. In our study, squamous cell carcinoma was the most common histology, accounting for 79.6%. Melanoma is classified under other histological types, but the number is unknown by this registration system. The majority of vaginal cancer was stage I. It is also consistent with previously described research [4, 14]. Most of the available data on treatment outcomes come from single institutions. While some studies report outcomes based on a single histological type or treatment modality, others include patient cohorts from different eras who received different treatments. The reported 5-year survival in larger series range from 64 to 84% for stage I, 53 to 75% for stage II, 36 to 53% for stage III, and 3 to 36% for stage IV [4, 15–20]. In our study, 5 year OS were 87.5% for stage I, 55.6% for stage II, 59.9% for stage III, and 15.3% for stage IV.

Due to the small number of patients followed for 5 years, we examined factors affecting prognosis based on the 3-year survival rate. Univariate analysis showed that FIGO stages were related to the prognosis, and that chemotherapy as primary treatment was associated with significantly poor prognosis. Multivariate analysis suggested that FIGO stage and age ≥ 80 years were the independent prognostic factors, and that surgery demonstrated a significantly better prognosis compared to chemotherapy and radiation therapy. However, analysis matching disease stages showed no statistical advantage for surgery. Furthermore, selection bias cannot be ruled out. Patients suitable for complete resection and younger patients with good general health condition and low risk of surgical complications are more likely to be selected for surgery, and such patients generally have a higher likelihood of a favorable prognosis. The surgery group was younger than the non-surgery group. In stage I, the median age in the surgery group was 51.5 years, whereas that in the non-surgical group was 62.5 years (*p* < 0.001, Wilcoxon rank sum test). On the other hand, elderly patients (age ≥ 65) who underwent surgery did not show a worse prognosis compared to younger patients (age < 65). Due to the inability to obtain data on tumor size, complications, and other factors, the details of how treatment decisions were made remain unclear.

This study is consistent with the results of various previous reports examining prognostic factors for vaginal cancer, enhancing the reliability of our findings. As with other malignancies, disease stage is recognized as the most important factor determining prognosis in vaginal cancer patients [18, 21, 22]. Higher stage may be associated with more comorbidities and reduced likelihood of surgery and thus lead to lower survival rates. Surgical therapy may be considered in clinical stages I and II if the tumor is located in the upper third of the vagina [23]. Most vaginal cancers occur in the upper third of the vagina, and about half of those are located in the posterior wall [7]. If a tumor extends to the middle or lower third of the vagina, it will probably require pelvic exenteration or vulvectomy to remove it completely. Because such invasive surgery significantly impairs quality of life, physicians tend to opt for radiation therapy. For stage I or II tumors of the upper vagina, as with cervical cancer, radical or modified radical hysterectomy combined with vaginal resection with adequate margins is often selected. A retrospective examination of 100 cases of primary vaginal cancer at a single institution reported that surgical treatment was associated with a better prognosis than radiotherapy alone when the tumor was located in the upper third of the vagina and was clinically stage I or II [24]. Similarly, in this study, surgery was significantly associated with a better prognosis compared to radiation, and no significant difference in prognosis was observed between the two age groups (< 65 years vs*.* ≥ 65 years). Additionally, although there was no statistically significant difference, the group requiring adjuvant radiotherapy had a tendency toward a poorer prognosis compared to the surgery-only group. In cases requiring additional treatment, surgical intervention may not improve prognosis. These findings suggested that surgery may be an effective option in early-stage vaginal cancer when cases are appropriately selected.

The introduction of CCRT for vaginal cancer treatment is based on data extrapolated from patients with locally advanced cervical cancer. A Cochrane review demonstrated that CCRT reduced the absolute risk of death by 6% and provided an 8% absolute benefit in disease-free survival in cervical cancer patients [25]. Due to the low incidence of vaginal cancer, conducting randomized trials is challenging. However, a large-scale study using the US National Cancer Database (NCDB) demonstrated that CCRT is an independent prognostic factor for improved overall survival (56 months in the CCRT group vs*.* 41 months in the radiotherapy group [26]. The most commonly used regimen is cisplatin 40 mg/m^2^ weekly, although other agents and combination therapies have also shown benefit [27–29].

In the treatment of vaginal cancer, brachytherapy is an essential component. Brachytherapy involves placing a radioactive source (usually Ir-192) directly into or near the residual lesion to deliver treatment. This allows for the delivery of an additional dose to the residual lesion while minimizing effects on normal tissue. Two large-database studies reported that discontinuing brachytherapy in vaginal cancer treatment leads to reduced survival rates. A large-database study published by the US Surveillance, Epidemiology, and End Results (SEER) Program found that the median overall survival period for vaginal cancer patients receiving brachytherapy (6.1 years) was over 2 years longer than for patients who did not receive the therapy (3.6 years) [30]. Multivariate analysis showed that the survival benefit from brachytherapy was independent of FIGO stage, tumor size, and histology. The effect of brachytherapy was most pronounced in patients with tumors > 5 cm [30]. Another study examining cases of vaginal cancer patients who received CCRT monotherapy using the NCDB database also found that additional radiation via brachytherapy was associated with improved 5-year survival rates (62.9% *vs*. 49.3%, *p* = 0.0126) [31].

This study did not record details of radiation therapy, and the effect of brachytherapy could not be evaluated.

Vaginal cancer is sometimes treated with chemotherapy in advanced or relapse cases, but evidence of its efficacy is scarce due to the small number of cases. Currently, it is used in conformity with the practice for cervical cancer [23].

Combination therapy with surgery and radiation therapy is also considered in the treatment of vaginal cancer. Patients with stage I or II vaginal cancer, who undergo surgical treatment, sometimes require adjuvant radiotherapy due to positive resection margins and/or positive lymph node metastasis. A study of 124 patients found that survival rates were equivalent between surgery alone and radiotherapy alone in stages I and II patients. However, 55% of patients received adjuvant radiotherapy after surgery [32]. In this study, 21 of 171 patients received radiation therapy after surgery, 20 received CCRT, and 26 received chemotherapy.

The limitations of our study should be noted. This study is retrospective in nature and is inherently subject to patient selection bias. Patients in better condition may have been selected for surgery. Several covariates of interest are unavailable from the JSGO database, including patients’ performance status and comorbidities, tumor size, tumor site, HPV status, metastatic site, and treatment parameters (surgical completeness, radiation dose, use of brachytherapy, chemotherapy regimen), which may influence the prognosis.

Due to the high proportion of elderly patients with vaginal cancer, long-term follow-up after treatment is often difficult, making it challenging to obtain long-term prognosis data. In fact, a total of 17.3% of patients were lost to follow-up in this study.

This study identified the trends in the treatment of vaginal cancer and prognosis in Japan. Vaginal cancer is rare and more likely to occur among older women. Steady and long-term data collection is needed for establishing future treatments.

## Supplementary Information

Below is the link to the electronic supplementary material.

Supplementary file1 (DOCX 4544 KB)

## Data Availability

Data are not publicly available in order to protect participant privacy.
