# Supplementary material for: Survival and prognostic factors in vaginal cancer: an analysis of 2016–2020 nationwide data
Source: Int J Clin Oncol. 2026 Apr 9;31(6):947–56. doi: 10.1007/s10147-026-03017-y (PMC13201297; doi:10.1007/s10147-026-03017-y)
Supplement: Supplementary file 1 — Supplementary file1 (DOCX 4544 KB) [file 10147_2026_3017_MOESM1_ESM.docx]

**Supplementary Figure 1** Survival curves by treatment for Stage I vaginal cancer


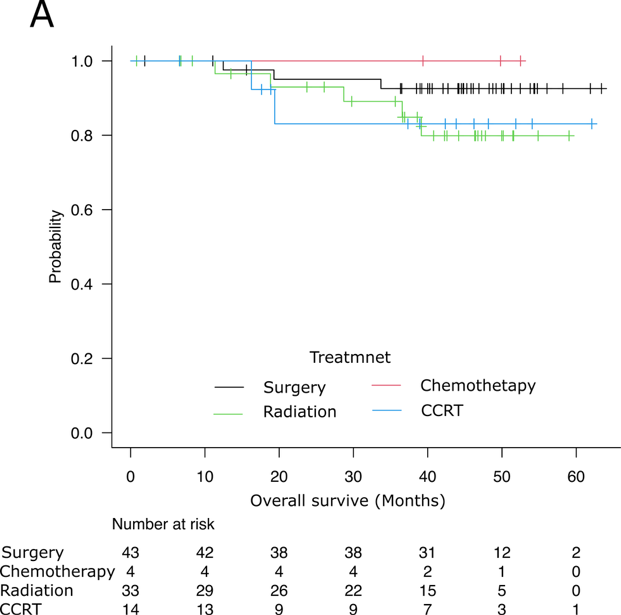

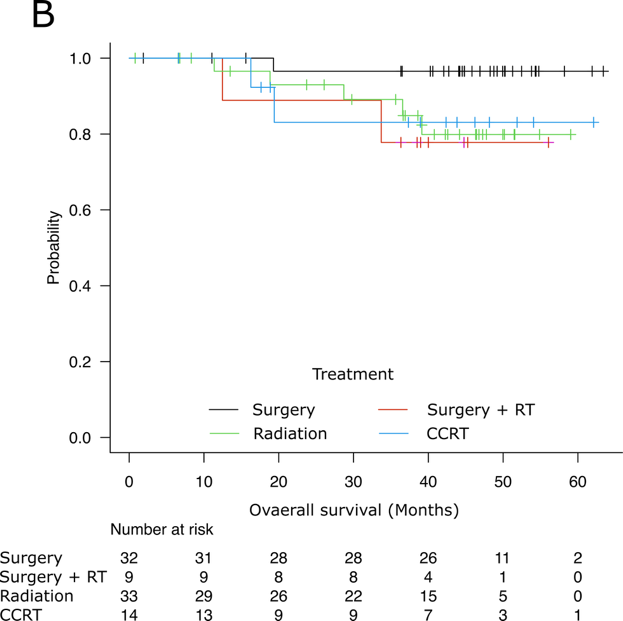


Overall survival curves by primary treatment methods (A). Surgery group was divided into surgery alone and surgery plus radiation therapy including CCRT (B). No significant differences were observed between any of the treatment groups.

**Supplementary Figure 2** Survival curves by treatment for Stage II vaginal cancer


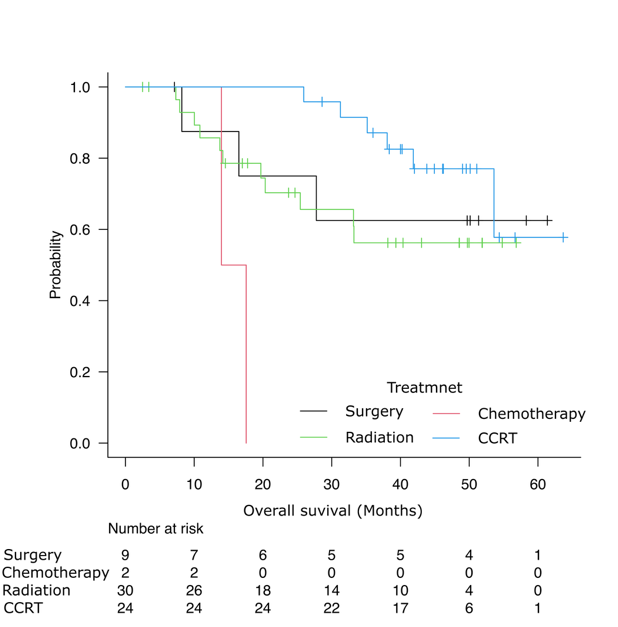


Overall survival curves by primary treatment methods. No significant differences were observed between any of the treatment groups.
